# Can hypnosis displace the threshold for visual consciousness?

**DOI:** 10.1093/nc/niy009

**Published:** 2018-11-20

**Authors:** Hernán Anlló, Jérôme Sackur

**Affiliations:** 1 Département d’Études Cognitives (École Normale Supérieure – PSL Research University), Laboratoire de Sciences Cognitives et Psycholinguistique (ENS, EHESS, CNRS), Paris, France; 2 Center for Interdisciplinary Research (CRI), Paris, France; 3 École des Hautes Études en Sciences Sociales, Paris, France; 4 École Polytechnique, Palaiseau, France

**Keywords:** hypnosis, hypnotic induction, posthypnotic suggestion, visuospatial attention, subjective visibility, semantic priming, cognitive control, task set

## Abstract

To test the specific effects of hypnosis on the attentional components of visual awareness, we developed a posthypnotic suggestion for peripheral visual inattention inspired on the “tunnel vision” symptom of the Balint Syndrome. We constructed a dual-target visibility and discrimination paradigm, in which single-digit numerical targets were placed both on the hypnotically affected peripheral space and on the remaining undisturbed central area. Results were 3-fold: (i) when compared to participants of Low hypnotic susceptibility (Lows), highly susceptible participants (Highs) presented decreased subjective visibility; (ii) Highs did not show dual-task interference from peripheral targets (an effect of unconscious processing) during hypnotic suggestion to not attend them, but Lows did; (iii) nevertheless, when asked to execute a discrimination task over these same targets, Highs performed with the same accuracy as Lows. These results suggest that the hypnotic manipulation of visuospatial attention did produce an experiential change in Highs, but not one that could be mapped onto interference at a single (conscious or unconscious) level of processing. Rather, we posit that Highs simultaneously displayed (i) a fluctuation in awareness of peripheral targets coherent with the suggestion and (ii) a control strategy that involved removing hypnotically unattended targets from the task set whenever task instructions would allow for it. In light of these findings, we argue that hypnosis cannot be used as a tool to restrict the processing of otherwise supraliminal stimulation to subliminal levels.

**Highlights**

- Hypnosis decreased subjective visual awareness for highly susceptible participants (Highs).
- However, Highs were still capable of discriminating between hypnotically unattended targets.
- Suggestion prevented unconscious processes elicited by hypnotically unattended supraliminal targets for Highs.
- Rather than rendering perception unconscious, we propose suggestion caused Highs to drop hypnotically unattended targets from the task set, but only when doing so didn’t conflict with task instructions.

## Introduction

Much has been written about the promising venues of hypnosis as a tool for cognitive research (Oakley and Halligan 2009, 2013; Raz 2011). In particular, recent reviews have proposed that hypnotic negative and positive hallucinations would be a valuable asset for the study of consciousness (Kihlstrom 2014; Landry *et al*. 2014). The rationale behind such affirmation stems mainly from the theoretical claim that hypnosis can alter percept consolidation by fostering a downplay of bottom-up perceptual information while simultaneously privileging the integration of endogenously generated features (Brown and Oakley 2004; Terhune *et al*. 2017). It has been argued that this top-down dismissal of perceptual information could potentially replace the physical modulation of stimulus energy, customarily used in the creation of preconscious and subliminal perception. It may thus constitute an alternative for the study of unconscious perception, one without the hindrances of physically degraded stimulation (Landry *et al*. 2014).

However, the exact psychological mechanisms by which hypnosis enacts this top-down control remain a matter of debate (Terhune *et al*. 2017). In recent years, some authors have proposed that hypnotic responding could be understood as the result of a particular instance of altered attention—more specifically, a form of top-down-driven “selective inattention” (Raz 2005, 2011; McLeod 2011; Lifshitz *et al*. 2012; Terhune *et al*. 2017). This notion originated from the seminal work of Raz *et al.*, in which the experimenters used a hypnotic alexia suggestion to successfully hamper the Stroop effect (Raz *et al*. 2005). They concluded that the obtained results implied a detour of the otherwise automatic attention allocation that supports both the reading process and the semantic processing of words (MacLeod 1991; Neely 1991).

While the aforementioned work generated new avenues of research pertaining the effects of hypnosis over automatic attention allocation and the cancellation/conciliation of cognitive conflict (Raz 2004; Raz *et al*. 2006; Terhune *et al*. 2010; Augustinova and Ferrand 2012), a finer-grained study of the effects of hypnotic suggestion, induction, and hypnotizability over the entire spectrum of cognitive mechanisms that compose attention remains direly needed. We have to date little knowledge as to how the concrete temporal and spatial dynamics of attention unravel during hypnotic responding, and the limits of how much (or how little) hypnosis can tamper with attentional resources are yet unclear (Terhune *et al*. 2017). These are questions with important ramifications, as models of consciousness such as the Global Neuronal Workspace (GNW) consider attention to play a key role in allowing stimuli into awareness (Dehaene *et al*. 2006).

In particular, a part of the experimental work sustaining the GNW account of consciousness has leveraged on spatial attention limitations, exploiting them in order to modulate awareness and task performance: paradigms aimed at eliciting unconscious perception would typically demand participants to fixate on the center of a blank screen while simultaneously requiring them to detect or discriminate peripheral targets. By adding peripheral masks and regulating peripheral stimulus onset, such paradigms could be attuned to test for task performance, priming effects, and both objective and subjective visibility in the periphery (Del Cul *et al*. 2006, 2007, 2009; Reuter *et al*. 2007, but see also Thibault *et al*. 2016). The rationale behind this manipulations relied on the fact that spatial attention capacity for selective improvement of processing is limited by its own resolution (Intriligator and Cavanagh 2001). This is particularly the case in parafoveal and extrafoveal areas, where attentional resolution drops as the targets distance from the fovea increases.

Uncovering therefore to what extent hypnosis can constrain or expand spatial attention selectively would constitute a fundamental step in the process of establishing how hypnotic suggestion may prevent otherwise supraliminal stimuli from becoming conscious. In this vein, the present work has specifically targeted visuospatial attention through posthypnotic suggestion, with the purpose of hampering subjective visibility and probing the extent to which hypnotically unattended information could be processed. We replaced masking and other forms of physically diminishing stimulus energy by our posthypnotic suggestion: a hypnotic instruction to not attend the periphery, inspired on the “tunnel vision” effect from the Balint Syndrome (Edgette and Edgette 1995). While of course our posthypnotic suggestion is not intended to exactly reproduce the pathology, pathology-inspired suggestions for the study of hypnotic visual attention are not unprecedented (Oakley and Halligan 2009, Supplementary Methods; Priftis *et al.* 2011).

Only a handful of studies to date have actually explored the workings of hypnotic inattention and its specificity when applied to visual awareness and subjective visibility. Efforts pursued to elicit full “hypnotic blindness” through suggestion (Bryant and McConkey 1989a,b, 1990) deserve a mention. Despite producing what highly hypnotizable subjects reported as the incapacity to see full-energy stimulation, and the clear potential such cognitive distortion could represent for the study of conscious awareness (Bryant and McConkey 1989b), by and large the existing studies have not targeted spatial attention specifically as the present work. Furthermore, participants have rarely been asked to perform a task dependent upon the blinded target, and while on some occasions objective measurements were taken (e.g. response times), the evaluation of the blindness itself has been mostly based on non-controlled subjective reports, which may be explained by non-hypnotic responses to demand characteristics (Mallard and Bryant 2001, 2006).

Through a “hemispatial neglect-inspired” hypnotic suggestion, Oakley and Halligan (2009) have managed to reproduce the symptoms of the hemineglect syndrome on a single “hypnosis virtuoso” participant but did not test for any kind of unconscious processing in the hypnotically neglected visual field (an ideal confirmatory measure, as it has already been shown that neglected spaces elicit different levels of unconscious processing; see Sackur *et al*. 2008). Priftis *et al.* (2011) developed Oakley and Halligan’s idea further, and implemented a “visual neglect hypnotic suggestion” on a number of participants highly susceptible to hypnosis, by explicitly demanding them to direct their visuospatial attention to only one side of their visual space. By having the participants perform a simple detection task while undergoing suggested posthypnotic effects, their results pointed out the neglecting of stimuli in the opposite side of the attended space: while very far from actual blindness or total lack of awareness, Priftis *et al.*’s participants did show significantly slower response times for the neglected stimuli. The Oakley and Halligan’s study was clinically inspired and evaluated subjective visual awareness only through broad phenomenological tests. On the other hand, Priftis *et al.*’s work did not directly test visual awareness but used response time as a proxy instead.

On a more recent note, Schmidt *et al.* (2017) implemented a slight variation of hypnotic blindness paradigms by hypnotically suggesting to their participants that their visual field was obstructed by a wooden board. Participants then wore an EEG net and were asked to solve an oddball task while under the influence of this suggestion, and while their actual visibility was not impaired, highly susceptible individuals showed a negative mean difference of 20% in performance. Furthermore, while Highs did not show any significant changes in the early physiological components typically associated with this kind of task (N1, P2), they did show a reduced P3b amplitude.

However, none of the previous studies considered if the unfolding sequence of processing that in normal circumstances progresses from unconscious to conscious mental activity was somehow prevented by hypnotic suggestion, causing perception to stop before emergence of awareness and if so does it continue to unfold unconsciously. Furthermore, they did not differentiate between the effects of induction and suggestion as distinct components of hypnosis. This differentiation, crucial for the correct understanding of hypnotic response and addressed by only a handful of studies, is fundamental inasmuch as existing evidence for the role of induction in suggestion-specific effects is preliminary at best (Terhune and Cardeña 2016). In the present work, we displayed peripheral targets at five different fixed durations (0 for a control baseline, i.e. no target, 16, 33, 67, and 84 ms) to probe for their visibility at various levels of stimulus energy. The core of this experiment was designed along the lines of classical hypnotic manipulations, i.e. a contrast between groups of High and Low hypnotic susceptibility. However, as an additional measure, we have proceeded to recruit a second group of highly susceptible participants and had them perform the experiment under the effect of the same suggestion, but in the absence of hypnotic induction [The decision of focusing on highly hypnotizable participants alone for this manipulation stemmed from two particular reasons. Firstly, because of the nature of hypnotizability measurements: hypnotizability scoring attributes the lowest grades to individuals who show little to no response in the face of several different types of hypnotic suggestion already within the context of an hypnotic induction (Shor *et al*. 1962; Anlló *et al.* 2017). Hence, we deemed it unlikely that Low hypnotizability participants would provide us with a richer, contrastable hypnotic response in the absence of induction. Secondly, because of highly susceptible individuals’ responsiveness: indeed, one of the main reasons why the relevance of hypnotic induction has been put into question has been the existence of experiments in which highly susceptible participants have reacted to suggestion in the absence of induction (see Terhune *et al*. 2016 for a review; see Raz *et al.* 2006; Augustinova and Ferrand 2012 as examples)]. We decided to perform this additional step in order to establish what, if any, where the palpable differences elicited by hypnotic induction in highly susceptible participants, whose response has already been argued to be potentially independent of the latter, and more likely linked to susceptibility.

## Materials and procedures

### Stimuli, task structure

Each stimulus consisted of a single black digit (2, 4, 7, and 9) of 0.8° of size on a uniform gray background (24.6 cd/m^2^). Stimuli were displayed in dark gray (18.4 cd/m^2^) when presented as the central target, and in lighter gray (21.4 cd/m^2^) when presented as peripheral targets, yielding respective Weber contrasts of −0.25 and −0.13. A central elliptical hollow placeholder (2°× 1.8°) was displayed in black. Four dot-shaped gray pointers were set in each quadrant at a distance of 4° from fixation, at the positions of potential peripheral targets (The value of gray utilized for peripheral stimuli and pointers derived of a pilot study featuring the same task as on the third block of the main study, but at multiple contrasts and durations. We used the method of constant stimuli to determine that peripheral targets of -.13 contrast would yield mean 71% accuracy across participants for the categorization task with a target duration of 67 ms. For further detail, refer to Supplementary Fig. A). All stimulation was prepared and displayed with the Psychophysics Toolbox for Matlab (Brainard 1997; Pelli 1997; Kleiner *et al*. 2007).

**Figure 1.**
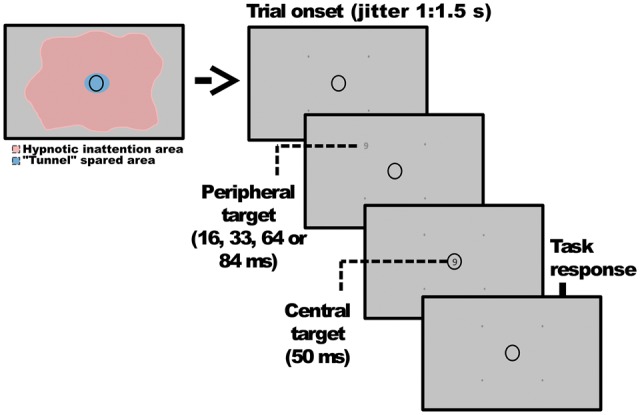
Graphical outline of the trial structure. All trials presented an identical structure, consisting of a Peripheral Target of variable duration (0 for a control baseline, i.e. no target, 16, 33, 67, and 84 ms) presented at either one of the four pointers set around the center of the screen, immediately followed by a Central Target of fixed duration (50 ms) displayed inside of the central ellipse. Only the task changed across blocks. Block order was balanced across participants.

All trials presented an identical structure (see Fig. 1), consisting of a peripheral target of variable duration (0 for a control baseline, i.e. no target, 16, 33, 67, and 84 ms) displayed after a random jitter of between 1 and 1.5 s from trial onset, at either one of the four pointers, and immediately followed by a central target of fixed duration (50 ms) displayed at the center of a central ellipse. Importantly, this central ellipse was defined during the posthypnotic and non-hypnotic suggestions as a space free of all inattention effects, “inside of the tunnel” (see Supplementary Methods for the full suggestion procedure). Immediately after stimuli presentation, the central ellipse flickered briefly to indicate that a response was expected. The trials were split into four blocks of 140 stimuli, with stimuli identity, duration, and position fully balanced within blocks. The task changed from block to block: in Block OC (Objective Central task), participants had to perform a discrimination task assessing if the Central Target was either greater or smaller than 5, by pressing the L or the M key on a standard AZERTY French keyboard with their right hand (relabeled for clarity). This block was conceived to test any possible priming elicited by the peripheral stimulus, as well as to test if hypnosis had any unintended effects on either accuracy or response times for the hypnotically “spared” targets. In Block SP (Subjective Peripheral task), participants were asked to evaluate the visibility of the Peripheral Targets through a perceptual awareness scale (PAS, Ramsøy and Overgaard 2004) ranging from 1 to 4, using their left hand on the Q, S, D and F keys (relabeled for clarity): “1” represented no experience of visibility, “2” a brief non-specific glimpse, “3” an almost clear experience of visibility, and “4” full visibility. This block was designed for evaluating peripheral subjective visibility, as a means for testing whether perceptual changes followed the content of the hypnotic suggestion. In Block OP (Objective Peripheral task), participants had to perform the discrimination task on the Peripheral Targets, with their right hand. The rationale behind this task was to test if highly susceptible participants were able to execute the task in a condition of reduced subjective visual awareness. Finally, in block OCSP (Objective Central task, Subjective Peripheral tasks), participants had to perform the discrimination task on the Central Target, and immediately afterwards, the Subjective Visibility task on the Peripheral Targets. This block was meant to test for congruency effects between peripheral and central targets when both were task-relevant and attended. Additionally, this block was designed to test if by paying attention at the same time to peripheral targets (which are affected by the posthypnotic suggestion) and central targets (spared by the posthypnotic suggestion), we would observe any hypnotic spillover effects over the central task.

### Participants

Participation was voluntary, in exchange of 15 € for a 1-h 30-min session. Participants were all contacted by e-mail and recruited by a research assistant independent to the study, from a database of volunteers previously screened with the French Norms of the HGSHS: A (Shor and Orne 1962; Anlló *et al*. 2017). Participants intervening in the High vs. Low hypnotic susceptibility contrast were told that they would take part of an experiment that would include their response to hypnotic suggestion and advised since first contact that all levels of susceptibility were equally relevant, equally important, and equally desirable for the experimenters. In the case of participants recruited for the No Induction group, no mention of hypnosis was made at all at during the procedure or at any point of the recruitment process.

A total of 24 right-handed, native French speakers aged between 19 and 35 (mean 24.8, SD: 3.41, 19 female) participated in the main contrast (High vs. Low hypnotic susceptibility): 12 participants highly susceptible to hypnosis (Harvard score 8–12, mean score 10, SD: 1), 12 of Low susceptibility (score 0–4, mean score 1.3, SD: 1) [In the reference material, i.e. the French Norms for the HGSHS:A (Anlló *et al*. 2017), the top 44.3% of the sample scored 8 or above in the corrected scale of susceptibility, and the bottom 29.5% scored 4 or below. We decided to make sure that our results were not then a product of the hypnotizability spread, particularly in Study 2 where one could worry that the Induction effect could actually respond to an hypnotizability difference between both High groups. In order to do so, we reran our model for Study 2, but only selecting the participants of both groups that had a Harvard score of 9 or 10 (5 participants for the No Induction group, 9 for the Induction group). Our results replicated the same differences observed in the original findings, all statistically significant (main Peripheral Target duration effect *P* < 0.0001, main Induction effect *P* < 0.0001, interaction between Peripheral Target duration and Induction *P* < 0.0001)]. Participants were called by an independent research assistant and tested in a random order, as to prevent the hypnosis practitioner from knowing their hypnotic susceptibility scores in advance.

For the Hypnotic Induction vs. No Hypnotic Induction contrast, an additional group of right-handed, native French speakers (mean age 24.3, 15 female) of all hypnotizability scores were recruited for preparing the No Induction control group. Participants of medium and High hypnotic susceptibility were summoned to participate of the experiments by an independent research assistant in a random order, as means of preventing the hypnosis practitioner from knowing about their hypnotic susceptibility. Once all experimental sessions were concluded, participant hypnotizability was made available to the first author, and the seven participants who presented High hypnotic susceptibility were retained for analysis as the No Induction group. Once all experimental sessions were concluded, participant hypnotizability was made available and the seven participants who presented High hypnotic susceptibility (mean age 24.14, SD: 4, 7 female, mean Harvard score 9, SD: 1) were retained in the No Induction group.

All participants signed a written consent allowing for the anonymous exploitation of the data they produced. The experiment was conducted in agreement with the Declaration of Helsinki (2008) and approved by the Ethics Committee of the Université Paris Descartes (Paris 5).

### Hypnotic induction and suggestion

The hypnotic induction consisted of a shortened variation of the gaze-fixation induction from the French Norms for the Harvard Group Scale of Hypnotic Susceptibility (Shor and Orne 1962; Anlló *et al*. 2017). The posthypnotic suggestion that ensued was based on the symptomatology of the Balint Syndrome (see Supplementary Methods for the full induction and suggestion procedures), and expressed in terms of attention, attention direction, and attentional modulation. The intended effect of this hypnotic procedure was to produce a visually unattended space outside of the central elliptical placeholder, ideally rendering “negligible” any stimuli present outside of this area. The first author, who is a licensed clinical hypnosis practitioner, constructed and administered both the induction and the suggestion while ignoring participant’s hypnotizability scores, but by design was not blind to the experimental condition (Induction or No Induction), as he was the experimenter and the hypnosis practitioner.

The suggestion for the No Induction control group was as similar as possible to the one implemented with hypnotized participants, as to elicit similar degrees of motivation and instruction, but without any hypnotic references. This suggestion was also administered by the first author, who was again blind to the hypnotizability of the participants until after the post-test interview. See Supplementary Methods for the full induction and suggestion procedures.

### Procedure

Participants sat in a dim-lit, soundproof test booth, equipped with a headset, a calibrated standard LCD screen, a chinrest fixed at 60 cm from the screen, at a height that assured that the participants’ resting gaze fell at the center of the screen. A standard keyboard for inputting responses was provided. Participants underwent then a Training phase consisting of a short version of each of the four blocks (25 trials per block). Crucially, after explaining the specific instructions for the blocks, participants were warned that at any given trial peripheral targets could be displayed “fast enough to seem completely absent,” but that a response was mandatory even if they felt like they were guessing. Participants were instructed to keep their gaze fixated on the center of the ellipse at all times, even when expected to perform a task on Peripheral Targets. Those who could not reach at least 90% accuracy on the OC task and 70% accuracy in the OP task, for durations of 67 and 84 ms, were to be discarded (none were). Participants were instructed to respond as fast as possible at all moments of the test, but never at the sake of their precision. Upon completion of the training phase, participants underwent the hypnotic induction and posthypnotic suggestion, or simply the suggestion, depending on the testing session. In order to trigger the posthypnotic suggestion into effect, the suggestion script stated that “as you return your head to the chinrest and fixate your gaze at the center of the ellipse, immediately your attention will focus on the inside of the ellipse and whatever happens inside of it, to the extent of rendering whatever may happen outside of it completely negligible, even invisible.” After suggestion delivery, the experimenter performed the scripted partial de-induction process, asked the participants to wear the designated audio headset, and left the room. Through the audio headset, participants were instructed by a recorded voice, clearly different from the experimenter’s, to adopt the position and place their head on the chinrest (as the experimenter verified through an obscured side window). Once in position, the recording announced the beginning of the experiment, explained the main tasks again and introduced each block as it came by repeating its instructions. Participants had to acknowledge proper understanding of the recorded instructions by pressing the “H” key for the block to start, or could choose to listen to the instructions again by pressing the “J” key (both relabeled for clarity). After the experiment, participants were de-induced and told to regain their normal awareness, and then debriefed and casually asked to be honest about their hypnotic experience. None of them reported any faking or “forcing” of the suggestion effects.

## Results

### Statistical analyses

We performed data analysis using R (R Core Team 2014). Response times and accuracy were modeled by implementing (generalized) linear mixed models, with a random intercept per participant (lme4, Bates *et al.* 2015). We selected this approach because of its numerous advantages, the main one being that the implementation of random intercepts per participant would allow us to control for individual variability in hypnotic responding. Fitting via maximum likelihood estimation typically ensures optimal properties of the estimators (Agresti 2013). We performed significance tests by means of likelihood ratio test that compared our models to simpler models, in which the relevant predictor was removed (null model). This model comparison approach enables us to provide some evidence for null effects, in case the null model is shown to provide a better fit (see below).

We compared models including as predictors Hypnotizability (levels: High, Low), Peripheral Target Duration (levels: 0, 16, 33, 67, 84 ms), Congruency between Targets (levels: Congruous (both stimuli below or above 5), Incongruous), Hypnotizability Score (levels: 1 to 12), and Block Type (levels: OC, SP, OP, OCSP). Selection of the best model was performed through likelihood ratio tests and based on Bayesian Information Criterion (Pinheiro and Bates 2000; Bolker *et al*. 2009). For each analysis, only the effects based on the best models are reported. ANOVA tables were computed through Analysis of Deviance (Type II Wald χ^2^ test), and *post hoc* pairwise comparisons through Tukey contrasts of least-squares means (0.95 CI) (car and lsmeans R packages, Fox and Weisberg 2011 and Lenth 2016, respectively). For proving actual lack of effects for given contrasts, we calculated the Bayesian Information Criterion approximation to the Bayes Factor for the full and null models originally implicated in said contrasts [BIC approximation to BF, so that BF = exp((BICfull-BICnull)/2), see Wagenmakers (2007)].

For each analysis, the full list of tested models with their respective Bayesian Information Criterion (BIC) is provided as Supplementary Table ST1.

### Liminality of peripheral targets

We decided to consider as subliminal or not perceptible all Peripheral Target categories in which participants’ objective discrimination task performance and visibility scores would be indistinguishable from control baseline (i.e. absence of Peripheral Target). We found that under this definition only the 17 ms category could be considered as subliminal. We arrived to this conclusion first by constructing four different linear models with participants as random intercept: each of these models estimated Subjective Visibility as a function of Hypnotizability (Low/High) and Peripheral Target Duration and their interaction. Each of these models consisted of only the trials corresponding to the baseline condition (0 ms, i.e. absence of Peripheral Targets) and the trials of one of the remaining four levels of the Peripheral Target Duration factor (17, 33, 67, and 84 ms). Comparing all models to their respective null models (which did not consider Peripheral Target Duration as a predictor), we found that Peripheral Target duration had a significant influence on Visibility (*P* < 0.01) for all models except the one that considered trials of 0 ms and 17 ms. When computing the difference in Bayesian Information Criterion (BIC) between this model in particular and its null, we found that the model that did not consider Peripheral Target duration as a predictor had a smaller BIC and was strongly favored by the BF computation (ΔBIC = 15.4; BF = 1480).

We then proceeded to do the same for the Objective Peripheral block data, and found the same results, again setting apart the model that comprised the 0 ms and 17 ms durations. On these bases, we established 17 ms trials to be subliminal (“unseen” mean subjective experience, indistinguishable performance from control), and the rest as supraliminal.

### Subjective visibility in the periphery diminishes for High participants

**Figure 2.**
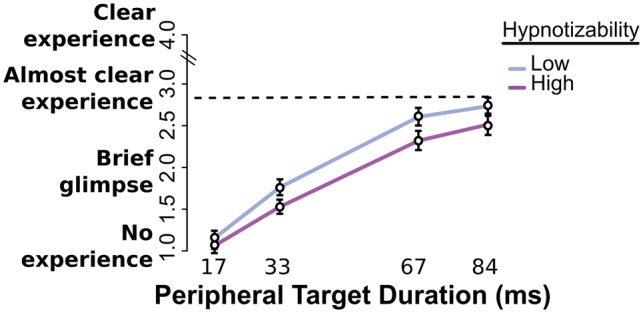
Visibility rating task. Subjective visibility (PAS scale) for all Peripheral Target durations and hypnotizabilities. Visibility rose globally with stimulus energy (*P* < 0.0001). Differences between hypnotizability groups increased with Peripheral target duration. SE bars calculated over grand mean minus participant mean, Morey-corrected.

Subjective visibility was measured through the implementation of a PAS ranging from 1 to 4, both in a single-task (block SP) and double-task framework (block OCSP). As displayed in Fig. 2, visibility increased globally with the rise in stimulus energy (Peripheral Target Duration main effect, χ^2^^ ^= 2904, DF = 3, *P* < 0.0001), but the *differences* in visibility between High and Low participants increased as a function of Target duration (Low Group > High Group; interaction Hypnotizability × Peripheral Target Duration χ^2^^ ^= 8, DF = 3, *P* < 0.05). These results fell within expectation, as they showed that the hampering effects of the posthypnotic suggestion were modulated coherently by hypnotizability. It should be noted however that this interaction did not hold (*P* > 0.08, BF = 40.7) if one looked only at the difference between 33 and 67 ms (near-threshold values), which could indicate that our effect appeared to be mainly due to an interaction with liminality. We tested the statistical significance of these effects by means of a regression with factors of Congruency, Peripheral Target Duration and Hypnotizability, over pooled SP and OCSP blocks, since evidence favored the model which lacked the Block factor (ΔBIC = 15). See full results in Table 1.

**Table 1.** Detection of peripheral targets

	χ^2^	Df	Pr(>χ^2^)
Visibility			
Hypnotizability	0.9	1	0.3
Peripheral Duration	2904	3	<0.0001
Congruency	6	1	<0.05
Hypnotizability × Peripheral Duration	8	3	<0.05
Hypnotizability × Congruency	0.3	1	0.6
Peripheral Duration × Congruency	2.7	3	0.4
Hypnotizability × Pdur × Congruency	2	3	0.6

Analysis of deviance (Type II wald χ^2^ test). Congruency, peripheral target duration, and hypnotizability as regressors; visibility as dependent variable. Crucially, results indicated a significant interaction between hypnosis × peripheral target duration, pointing to the fact that, for highly susceptible participants, the posthypnotic suggestion of peripheral inattention hampered subjective visibility more when stimulus energy was high.

**Figure 3.**
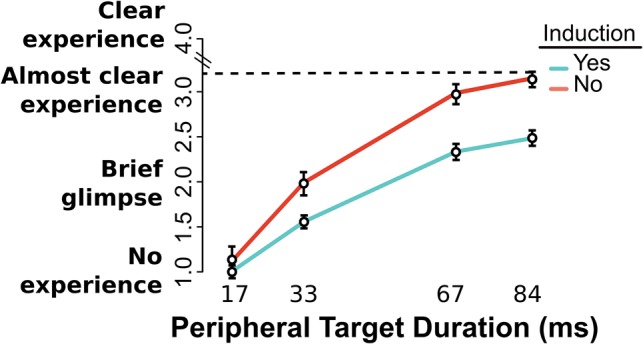
Visibility rating task. Subjective visibility (PAS scale) for all Peripheral Target durations, for highly susceptible participants with and without a hypnotic induction. Visibility was lower for those who went through the Induction process (*P* < 0.05) and increased with Peripheral Target duration (*P* < 0.0001). Visibility differences between Induction and No Induction groups increased significantly as a function of Peripheral Target Duration (*P* < 0.0001). SE bars calculated over grand mean minus participant mean, Morey-corrected.

The data collected from the No Induction testing session allowed us to evaluate the impact of hypnotic induction for highly susceptible participants. As displayed in Fig. 3, subjective visibility increased globally with stimulus energy (Peripheral Target Duration main effect, χ^2^^ ^= 3099, DF = 3, *P* < 0.0001) and decreased with Induction implementation (No Induction > Induction, Induction main effect χ^2^^ ^= 5, DF = 1, *P* < 0.05). We also observed that the difference in subjective visibility between Induction and No Induction groups increased as a function of increased stimulus energy (No Induction > Induction, interaction Induction implementation × Peripheral Target Duration χ^2^^ ^= 117, DF = 3, *P* < 0.0001). Overall, these findings point to the fact that the introduction of a hypnotic induction further hampered subjective visibility, even more so for high-energy stimuli. In this case, the interaction held even when only looking at the difference between 33 and 67 ms (near-threshold values, *P* < 0.001).

We tested the statistical significance of these effects by means of a regression with factors of Congruency, Peripheral Target Duration, and Induction implementation, over pooled SP and OCSP blocks. Full results can be found in Table 2.

**Table 2.** Detection of peripheral targets

	χ^2^	Df	Pr(>χ^2^)
Visibility			
Induction	5	1	<0.05
Peripheral Duration	3099	3	<0.0001
Congruency	4	1	0.06
Induction × Peripheral Duration	117	3	<0.0001
Induction × Congruency	0.03	1	0.9
Peripheral Duration × Congruency	5	3	0.2
Induction × Pdur × Congruency	3	3	0.5

Analysis of deviance (Type II wald χ^2^ test). Congruency, peripheral target duration, and induction implementation as regressors; visibility as dependent variable. These findings point to the fact that the introduction of a hypnotic induction further hampered subjective visibility, and even more so for high energy stimuli.

**Figure 4.**
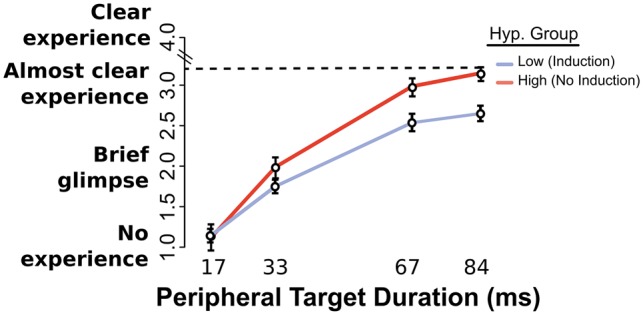
Visibility rating task. Subjective visibility (PAS scale) for all Peripheral Target durations, for highly susceptible participants who received a non-hypnotic suggestion and Low susceptibility participants who underwent the full hypnosis process. Visibility differences between both groups increased as a function of Peripheral Target duration (*P* < 0.0001). SE bars calculated over grand mean minus participant mean, Morey-corrected.

Faced with a significant visibility difference in favor of the No Induction High participants, we proceeded to compare their visibility judgements with those of the Induction Low participants from Study 1. As indicated in Fig. 4, as stimulus energy rose, the suggestion for visual inattention hindered the subjective awareness of High (No Induction) participants less than for Low (Induction) participants, despite their High hypnotic susceptibility [High (No Induction) > Low (Induction), interaction Hypnotizability × Peripheral Target Duration χ^2^^ ^= 64, DF = 3, *P* < 0.0001].

We tested the statistical significance of these effects by means of a regression with factors of Congruency, Peripheral Target Duration, and Hypnotizability [levels: High (No Induction), Low (Induction)], over pooled SP and OCSP blocks as with the original dataset. Full results can be found in Table 3.

**Table 3.** Detection of peripheral targets

	χ^2^	Df	Pr(>χ^2^)
Visibility			
Hypnotizability	1.5	1	0.2
Peripheral Duration	2987	3	<0.0001
Congruency	6	1	<0.05
Hypnotizability × Peripheral Duration	64	3	<0.0001
Hypnotizability × Congruency	0.1	1	0.8
Peripheral Duration × Congruency	6	3	0.1
Hypnotizability × Pdur × Congruency	1.6	3	0.7

Analysis of deviance (Type II wald χ^2^ test). Congruency, peripheral target duration, and hypnotizability [Highs (No induction), Lows(Induction)] as regressors; visibility as dependent variable. Results were consistent with a significant impact of induction implementation in reducing visibility.

### Suggestion impairs priming effects stemming from the periphery for High participants

**Figure 5.**
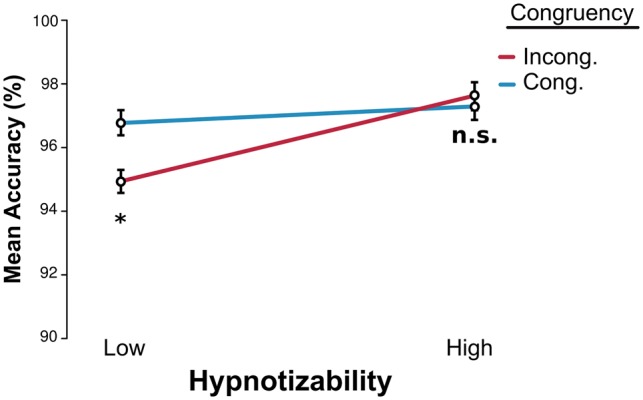
Central discrimination task. Accuracy scores (percentage correct) for congruent and incongruent trials, across Low and High susceptibility participants. Accuracy differences related to Congruency were larger for the Low group (*P* < 0.05). Accuracy remained unaffected by Congruency for the High group (*P* > 0.08, BF = 40.4 in favor of the null model; model on the High population alone). SE bars calculated over grand mean minus participant mean, Morey-corrected.

The question remained of whether hypnotic inattention was only active at the subjective level or if it impaired cognitive processing of stimuli. To answer this question, we turned to the investigation of priming effects between the peripheral and central stimuli. Because of trial structure, Peripheral Targets worked as primes for the Central Target in blocks OC and OCSP. As shown in Fig. 5, the accuracy difference between Congruent and Incongruent trials was significantly larger for Lows than for Highs (Congruent > Incongruent, interaction Hypnotizability × Congruency, χ^2^^ ^= 4, DF = 1, *P* < 0.05). Moreover, congruency did not appear to play a role at all in modulating accuracy for High participants (*P* > 0.08, BIC approximation to BF = 40.4 in favor of the null model). We tested the statistical significance of this effect by means of a binomial regression on accuracy with factors of Congruency and Hypnotizability, over pooled OC and OCSP blocks. Full results are in Table 4.

**Table 4.** Central discrimination task

	χ^2^	Df	Pr(>χ^2^)
Accuracy			
Hypnotizability	1	1	0.3
Congruency	2	1	0.1
Hypnotizability × Congruency	4	1	<0.05

Analysis of deviance (Type II wald χ^2^ test). Congruency, peripheral target duration, and hypnotizability as regressors; accuracy as dependent variable. Congruency did not appear to play a role at all in modulating accuracy for High participants (BIC approximation to BF = 40.4 in favor of the null model; model on the High population alone).

For further verification, we repeated the analysis utilizing raw hypnotizability scores of all participants as a regressor, rather than the Group Type factor, and replicated the result, i.e. difference in performance for incongruous and congruous trials increased in inverse relation to hypnotizability (Congruence × Individual Hypnotizability Score, χ^2^^ ^= 4, DF = 1, *P* < 0.05). Overall our results point to the fact that when processing the central stimulus, highly hypnotizable individuals seem to be shielded from the influence of the peripheral stimulus, contrary to Low hypnotizable individuals.

As for the Induction vs. No Induction contrast within High responders, as shown in Table 5, a binomial regression on accuracy with factors of Congruency and Induction implementation did not reflect any interactions or main effects. Furthermore, the BIC approximation to BF taking the Induction factor as a predictor of accuracy favored the null model decisively (BF = 3964). Given the small sample size of the High No Induction condition, and considering that the BIC “penalizes” more complex models when *n* < 8 (James *et al*. 2013), we also used the Akaike Information Criterion (AIC). The AIC also favored the null model as the better fit (ΔAIC = 4).

**Table 5.** Central discrimination task

	χ^2^	Df	Pr(>χ^2^)
Accuracy			
Induction	0.04	1	0.8
Peripheral Duration	1	1	0.4
Congruency	0.03	1	0.9

Analysis of deviance (Type II wald χ^2^ test). Congruency, peripheral target duration, and induction implementation as regressors; accuracy as dependent variable. The BIC approximation to BF taking the induction factor as a predictor of accuracy favored the null model decisively (BF = 3964), indicating that induction likely did not play a significant role modulating accuracy.

When considered together with the results from the previous contrast (Highs vs Lows under posthypnotic suggestion), the fact that hypnotic induction would have no effect of its own over congruency-related effects suggested that the shielding from the peripheral target depends on suggestion and hypnotic susceptibility alone. Additionally, the lack of impact of hypnotic induction on the accuracy of the central discrimination task implied that the general relaxation suggestions and mental imagery evoked by the induction procedure did not have any non-specific effects over the task that they were intended to spare.

### Posthypnotic suggestion did not impair discrimination of peripheral targets for High participants compared to Low participants

**Figure 6.**
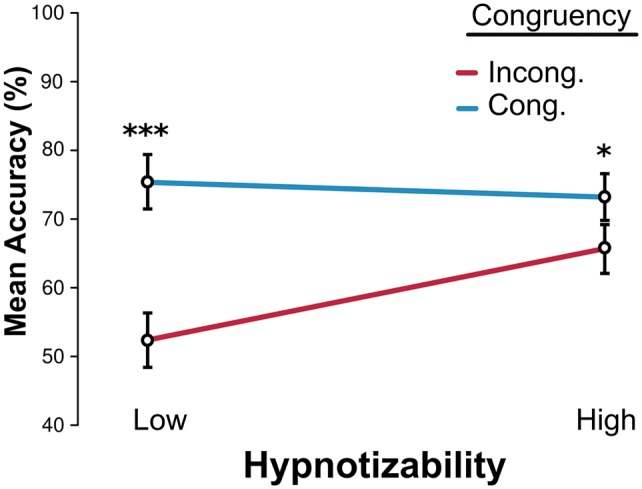
Peripheral discrimination task. Accuracy scores (percentage correct) for all collapsed Peripheral Target durations, across both groups of participants. Results showed a significant Interaction between Hypnosis × Congruency (*P* < 0.0001). SE bars calculated over grand mean minus participant mean, Morey-corrected.

In Study 1, we found that highly hypnotizable individuals responded to the attentional suggestion by manifesting a reduced subjective visibility of peripheral targets, as well as a lack of priming effects. In order to evaluate how these hypnotically induced changes impacted objective task performance, Block OP was designed to provide evidence on whether highly susceptible participants would be able to execute a discrimination task on these same peripheral, hypnotically unattended targets, on par with Low susceptibility participants. Results displayed in Fig. 6 show that irrespective of subjective awareness differences, High participants were nevertheless able to perform the task with better-than-chance accuracy. In addition, unlike blocks OCSP and OC, performance was significantly worse for incongruous trials also for highly susceptible participants (Congruent > Incongruent, main effect of Congruency, χ^2^^ ^= 71, DF = 1, *P* < 0.0001), even though the difference effect was indeed more pronounced for Low participants (interaction between Hypnosis × Congruency, χ^2^^ ^= 18, DF = 1, *P* < 0.0001; Tukey pairwise Congruency contrasts for Hypnotizability; High *P* < 0.05, Low *P* < 0.0001). We tested these effects with a model with Peripheral Target Duration, Congruency, and Hypnotizability as regressors for accuracy. Full results are in Table 6.

**Table 6.** Peripheral discrimination task

	χ^2^	Df	Pr(>χ^2^)
Accuracy			
Hypnotizability	2	1	0.2
Peripheral Duration	150	3	<0.0001
Congruency	71	1	<0.0001
Hypnotizability × Peripheral Duration	3	3	0.3
Hypnotizability × Congruency	18	1	<0.0001
Peripheral Duration × Congruency	1	3	0.8
Hypnotizability × Pdur × Congruency	3	3	0.4

Analysis of deviance (Type II Wald χ^2^ test). Congruency, peripheral target duration, and hypnotizability as regressors; accuracy as dependent variable. Results confirmed the interaction between the hypnotizability of each group and congruency (Tukey pairwise congruency contrasts for differences in accuracy across levels of hypnotizability: High, *P* < 0.05; Low, *P* < 0.0001).

## Discussion

We administered a posthypnotic suggestion for selective inattention to High and Low hypnotic susceptibility participants, inspired by the “tunnel vision” symptom of the Balint Syndrome. We set out to evaluate how the creation of an “unattended visual space” would successfully degrade subjective visibility and modulate information treatment at different levels of stimulus energy and hypnotizability. We did so through a single/double-task design, which asked for visibility judgments and target discrimination, and placed targets in the center of the visual field, outside the influence of the suggestion, and closer to its periphery, within the influence of the suggestion. This allowed us to both evaluate the efficacy and specificity of the posthypnotic suggestion, as well as its interference with any priming or congruency effects between peripheral and central targets. Additionally, we utilized the same paradigm, and set out to observe the same phenomena, but in connection to the specific influence of hypnotic induction on highly susceptible participants.

Our main findings are 3-fold. First, the contrast between High and Low susceptibility participants showed that our standard posthypnotic suggestion could hamper Highs’ subjective visibility (when compared to Lows’). This was particularly relevant, as it confirmed that the hypnotic procedure had successfully affected the participants’ visual experience in a way consistent with the content of the posthypnotic suggestion. We propose that such a difference in subjective visibility could be interpreted in terms of a difference in awareness, inasmuch as reportability and cognitive accessibility are regarded in many preponderant theories of consciousness as an index of awareness (Kouider 2009; Kouider *et al*. 2010). The actual nature of these changes in visual awareness, however, were not consistent with displacement of the perceptual threshold for access to consciousness (Del Cul *et al*. 2007), as there is no impact of hypnosis on subjective visibility at intermediate, near-threshold durations (33 and 67 ms, *P* > 0.08, BF = 40.7). Rather, we propose, they were the result of a belated, control-related process. Indeed, our results have shown that immediate basic perceptual processing did not depend of participant hypnotizability (with peripheral liminality being the same for all participant groups), and that this parity did not prevent the emergence of subjective visibility differences dependent of hypnotizability that hinged on the increase of stimulus energy. In other words, we posit that Highs found their awareness obscured by the posthypnotic suggestion only as stimulus energy rose and targets became perceptually accessible (supraliminal).

Additionally, we find this interaction to constitute as well a favorable argument against attributing the reported effects solely to expectations, or demand characteristics unrelated to hypnosis. Namely, if the Low hypnotizability group had approached the task with the expectation of responding to show a conserved full visibility, these participants would have likely overestimated visibility for lower stimulus energy targets. Conversely, if the High hypnotizability group had approached the task with the opposite expectation, then visibility would be expected to plateau instead of continuing to rise with increasing stimulus energy. Finally, both the thesis for a late, control-related effect and the argument against solely non-hypnotic effects of demand characteristics, were strengthened by the fact that our results showed hypnotic induction to extend this diminishment of visibility *also* in relation to stimulus energy.

It should be noted at this point that many early studies on the nature of hypnotic induction have found the latter to bear little to no impact on hypnotic responding (Hilgard and Tart 1966; Braffman and Kirsch 1999). Our findings, on the other hand, support the opposing conclusion. Results from the present work have shown that highly susceptible individuals who went through a non-hypnotic suggestion manifested a higher subjective visibility score than Low susceptibility participants who underwent hypnotic induction and hypnotic suggestion. We consider this to be a relevant contribution concerning the study of hypnotic induction, one far from being particularly odd in light of recent discoveries. First, several authors consider that the traditional definition of Low susceptibility individuals as people impervious to hypnosis and paragon of non-hypnotic normality could be the result of confirmation bias. To quote a recent review on new perspectives of hypnosis research, by Jensen *et al*. (2017, page 6): “In studies comparing lows and highs (e.g. Horton *et al.* 2004), the findings cannot always be properly interpreted; for example, it is not possible to determine if any between-group differences found are due to highs being atypical or lows being atypical (Lynn *et al.* 2007).” Secondly, clinical studies have shown that when given the proper motivation and the right set of expectations [which, incidentally, is for some the main role of hypnotic induction (see Terhune *et al*. 2017 for a review)] participants considered as Low could respond to hypnotic suggestion (Orne *et al.* 1996). Finally, recent neuroimaging studies have shown that particularly when it comes to suggested phenomenological changes, induction can play a major role in hypnotic responding, and that its distinctive and specific effects are traceable in the brain (Derbyshire *et al.* 2009; McGeown *et al*. 2012). Regrettably, such a performance on behalf of the Low group shows that a design including a contrast between Induced and Non-Induced Lows would have been more fruitful for a better understanding of the actual role of Induction.

The second set of main findings stemming from our results concern the impact of our posthypnotic suggestion on priming. Our results have shown that highly hypnotizable participants remained impervious to the influence of incongruent primes in the periphery for the central discrimination task, while the Low hypnotic susceptibility group did not. These results expanded our understanding of the specific information treatment caused by hypnotic inattention, particularly in connection to the question of whether utilizing hypnosis as a device would warrant subliminal or preconscious processing (Landry *et al*. 2014). The reported reduced subjective awareness of peripheral primes manifested by the High group would have been promising in this precise sense if it had also elicited any unconscious stimulus treatment, comparable to that of subliminal or pre-attentive stimulation (e.g. strong priming effects stemming from the hypnotically affected periphery for highly susceptible participants, paired with across-the-board reduced subjective visibility). Yet, our results showed that, at least within the context of this paradigm, highly susceptible participants disregarded hypnotically unattended peripheral information and did not use it, preventing it from influencing the central target task. While these results may discourage the idea of implementing hypnotic suggestions as a replacement for physical stimuli suppression techniques such as masking, they do however contribute to the literature questioning the automaticity of priming effects and the latter’s susceptibility to cognitive control (Kunde 2003; Kunde *et al*. 2003; Kiesel *et al.* 2005; Kiesel *et al.* 2007; Kiefer *et al.* 2012). While classical theories of automaticity assume that automatic processes elicited by unconscious stimuli are autonomous and independent of higher-level cognitive influences, the aforementioned findings bring forward evidence that unconscious visual processing is automatic only in the sense that it is initiated without deliberate intention, but susceptible to attentional top-down control and only elicited if the cognitive system is “configured accordingly” (Most *et al*. 2005; Kiefer *et al*. 2012). Attentional influences on priming depend not only on attentional resources but can also be modulated through stimulus expectations, intentions, and task sets (Kiefer *et al*. 2012). We suggest that posthypnotic-induced inattention attenuated priming processes stemming from the periphery for highly susceptible individuals as a result of a late, high-order manipulation, likely originated at the level of cognitive control. Rather than producing a perceptual lack of vision, the posthypnotic suggestion led highly susceptible participants to actively (albeit not purposefully) execute the task of “not seeing” through the top-down process of dis-attending to selected information. Results from the induction contrast reinforced this idea: the fact that priming effects were sensitive to hypnotic susceptibility but not to induction could point to the fact that, for highly hypnotizable individuals, direct suggestion is sufficient, and probably bears the same weight as task instructions when it comes to top-down designation of relevant information and its unconscious processing. This idea could also explain why hypnotic induction failed to have an influence over accuracy for the blocks OC and OCSP, but did alter the posthypnotic effects in subjective visibility for blocks SP and OCSP. Designating the relevance of peripheral stimuli could be conceived as a binary judgment (either relevant or irrelevant) that can be biased sufficiently through suggestion alone (before even starting the task, at suggestion). Visibility, on the other hand, was a gradual judgment to be established *in situ* (during each trial) amidst the contradiction between the suggestion, instructional content and variable physical energy. We think that in this condition, participants were more likely to be biased by the cumulative motivational and phenomenal changes warranted by the induction.

The third and last main set of findings from this study pertain to the objective discrimination task over hypnotically affected peripheral targets. Our results have shown that when probed by an objective peripheral task, both High and Low participants were able to perform the task despite the suggestion, and that they were vulnerable to backward contaminating information from the central stimulus. This finding is of relevance (i) because it confirmed the specificity of the posthypnotic suggestion, as the suggestion did not prescribe any restrictive effects for the central targets and (ii) because the extent to which High and Low participants were under the contaminating influence of the central stimulus was different. The fact that the performance differences between congruent and incongruent trials were smaller for highly susceptible participants was consistent with the growing body of literature identifying highly susceptible participants as better at reducing cognitive conflict (Raz *et al*. 2005, 2006; see Terhune *et al*. 2017 for a review), or rather, would suggest that highly susceptible participants as better at optimizing response production in the face of cognitive conflict [We find this second approach much more precise, as it allows us to acknowledge findings such as those by Egner *et al.* (2005), where highly susceptible participants showed higher activation of the dorsolateral ACC, known as an important part of the conflict network (when compared to Low susceptibility). Hence, what we propose is not that cognitive conflict is reduced *per se*, but rather, dealt with more efficiently. We posit that dACC increased activity would be a reflection of this improved conflict resolution scheme for highly susceptible individuals]. Indeed, by being able to better reconcile the contradictory/incongruent influence of the central stimulus over the peripheral target information, highly susceptible participants outperformed Low susceptibility participants on incongruent trials. A final point of importance to consider was the fact that (iii) Highs managed to perform on par with Low participants for congruent trials altogether, despite simultaneously reporting lesser visibility for the subjective visibility task. A weak interpretation of these results would imply that the suggestion managed to reduce subjective visibility, but not enough to have a real impact over objective performance. A stronger interpretation would imply that since the task was of the forced-choice variety, Highs’ performance in the face of reduced awareness was supplemented by the unaware treatment of the “hypnotically less-attended” peripheral targets. Post-session interviews tended to support the stronger view, with highly susceptible participants invariably reporting the impression of seeing “next-to-nothing” in the periphery, and manifesting little to no confidence regarding their performance over peripheral targets.

In all, these findings provide a consistent picture of the effects of hypnotic inattention as a tool for hampering subjective visibility and cognitive processing in a top-down fashion. In particular, they have allowed us to identify and separate the mechanisms by which posthypnotic induction and suggestion hampered visual awareness and reduced cognitive conflict. One mechanism, susceptible to the influence of hypnotic induction and dependent on hypnotizability that intervened belatedly into reshaping the subjective awareness of affected stimuli and was all the more present the higher stimulus energy was. And another top-down mechanism, potentially impervious to induction, dependent on the instructional content of suggestion, similar (if not homologous) to task instructions set, that mediated the attribution of relevance to certain segments of the visual space for particular tasks in a way that was congruous with both the suggested hypnotic effects and the task demands. Crucially, this last mechanism did not render stimuli unconscious. Instead, we propose it preemptively affected peripheral target processing: whenever task instructions did not call for these targets explicitly, Highs dropped them from the task set as per suggestion content (Most *et al.* 2005; Kiefer *et al*. 2012). This same results attest to the cognitive flexibility of highly susceptible participants, at both handling incongruent semantic information and the conflict elicited between suggestion (e.g. “ignore the target”) and task instructions (e.g. “perform a task over the target”), by always privileging performance.

Finally, it should be mentioned that our results could arguably limit the possibilities of implementing hypnosis as a classic subliminal masking tool, as purported by some theoretical reviews in the field (Kihlstrom 2014; Landry *et al*. 2014). Rather than physically causing perception to stop or deviate before emergence of awareness, hypnotic perceptual, and cognitive alterations respond to a balance between suggestion, expectation, and task instructions, leading the highly hypnotizable individual to integrate the three in the form of high-order regulations that privilege conflict reduction. Hypnosis does not seem like a suitable tool for simply restricting the processing of an otherwise supraliminal stimulus to the subliminal level. The more susceptible individuals may be, the more they may be able to flexibly adapt to suggestion and task instructions, giving rise to effects phenomenologically similar to traditional masking, but clearly not the same, as suggestion has been demonstrated here to interfere with the unconscious processing that normally precedes or accompanies conscious awareness of the target stimulus.

The effect of hypnotic suggestion over performance worked through top-down control; more precisely, we propose, through the impact that suggestion had over the task attentional set. Indeed, our posthypnotically induced spatial inattention experiment showed that while certain hypnotic components such as hypnotic induction had a strong effect over late subjective markers (such as visual awareness), low-level automatic processing could only be altered *a priori* and indirectly, through the kind of broad, goal-oriented high-order control usually attributed to cognitive strategy. The lack of semantic priming stemming from the hypnotically unattended targets strengthened this idea: rather than having a direct incidence on sensory processing (as physical masking does), the posthypnotic suggestion fostered the strategic dismissal of the perceptual information.

With this fact in mind, we could very prudently posit a rudimental cognitive model of hypnotic responding as a two-tier process. On one tier, the suggestion would actualize the task set, provoking attention to be driven towards the hypnotically targeted components. We posit that this would happen independently of what the actual instruction may consist of, independently of how much it could conflict with the pre-existing representational set or the original task instructions, and independently of hypnotic susceptibility. This could explain why, when probed on a task other than peripheral visibility over hypnotically affected targets, High participants showed such high performances, despite their subjective visibility scores. On the other tier, with a variable degree of involuntariness, participants would attempt to manage this new updated task set, which would often hold a contradiction between suggestion content and the original attentional set. Those participants we commonly identify as highly susceptible individuals would be able to privilege the suggested new task set components, and avoid contradiction by updating their cognitive regulation and disengaging from resource-intensive conflict monitoring. Crucially, we propose that they will do so under the umbrella of a subjective phenomenological experience that reflects suggestion content.

## Data availability

Data are licensed for non-commercial use only and have been made publicly available for download at the OSF repository of the project (https://osf.io/b6ry9/? view_onl =2264b3a032bc44358a43bb4e45fc0aa). Please address any questions to the corresponding author at hernan.anllo@cri-paris.org.

## Supplementary Material

Supplementary Data
