# Supplementary material for: Can hypnosis displace the threshold for visual consciousness?
Source: Neurosci Conscious. 2018 Nov 20;2018(1):niy009. doi: 10.1093/nc/niy009 (PMC6247368; doi:10.1093/nc/niy009)
Supplement: Supplementary Data [file niy009_supplementary_data.docx]

**2.9 SUPPLEMENTARY MATERIALS**

**2.9.1 Annex A: Additional Plots & Model Comparisons**


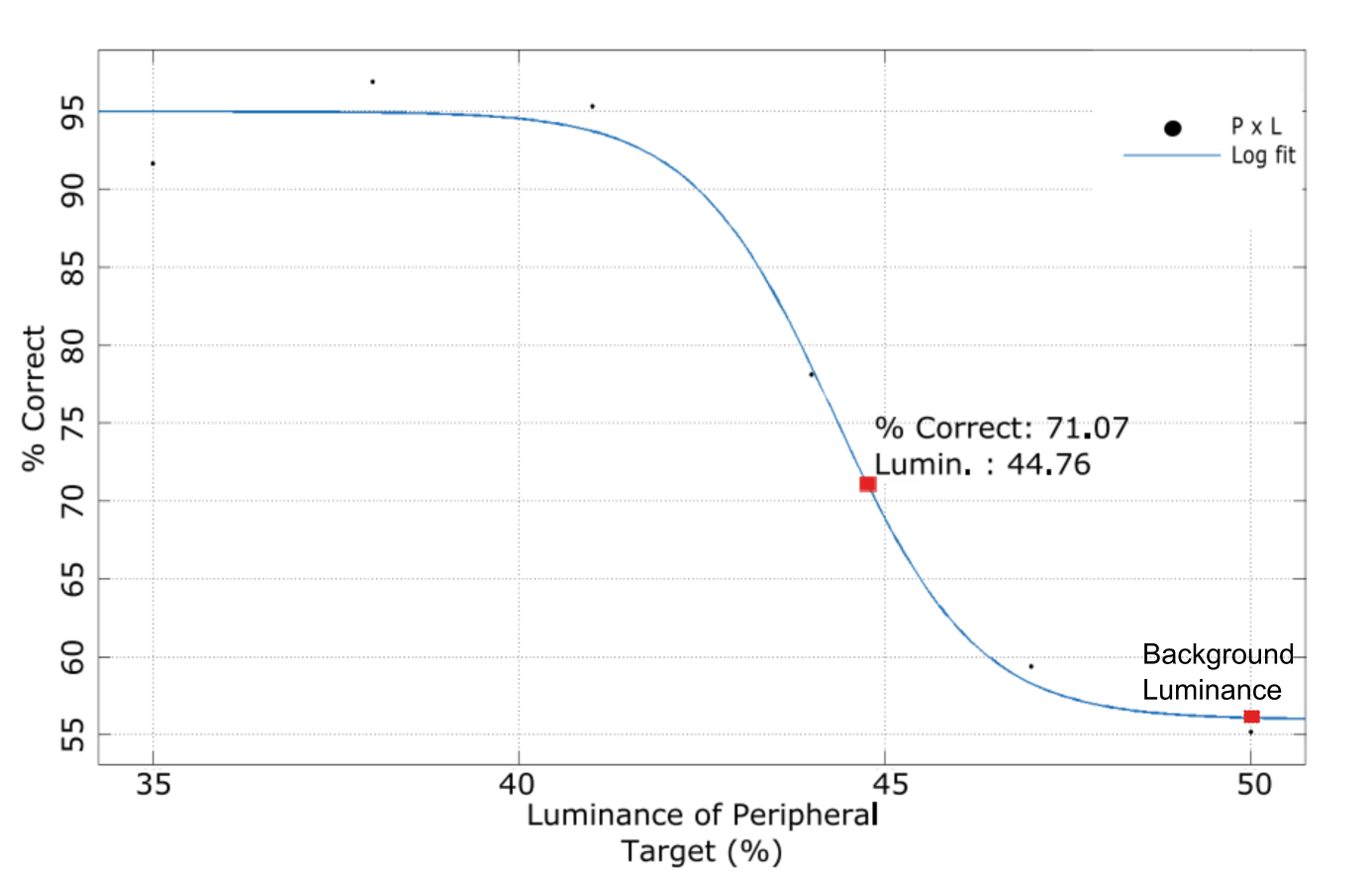


**Figure A. Psychophysical curve fitted for the establishment of the Peripheral Target luminance value.**

We performed a visual inspection of the results of a first pilot study, in which 6 subjects performed the task of Block 3 at fixed a luminance value (gray 60% CMYK scale) at 9 randomized display durations (0, 16, 33, 50, 67, 84, 100, 130, 150, ms). We established 67 ms to be the first stable supraliminal duration after the performance raise of the sigmoidal psychophysical curve. We then did a second pilot study with a separate group of 8 participants, in which we fixated the duration of the Peripheral Target to 67 ms, and tested performance for 4 different luminance levels (35%, 40%, 45% and 50% gray CMYK scale). A logistic fit of the resulting data allowed us to estimate a value of gray 44.76% on the CMYK scale for a predicted performance of 71% for durations of 67 ms.

**Table ST 1**

**
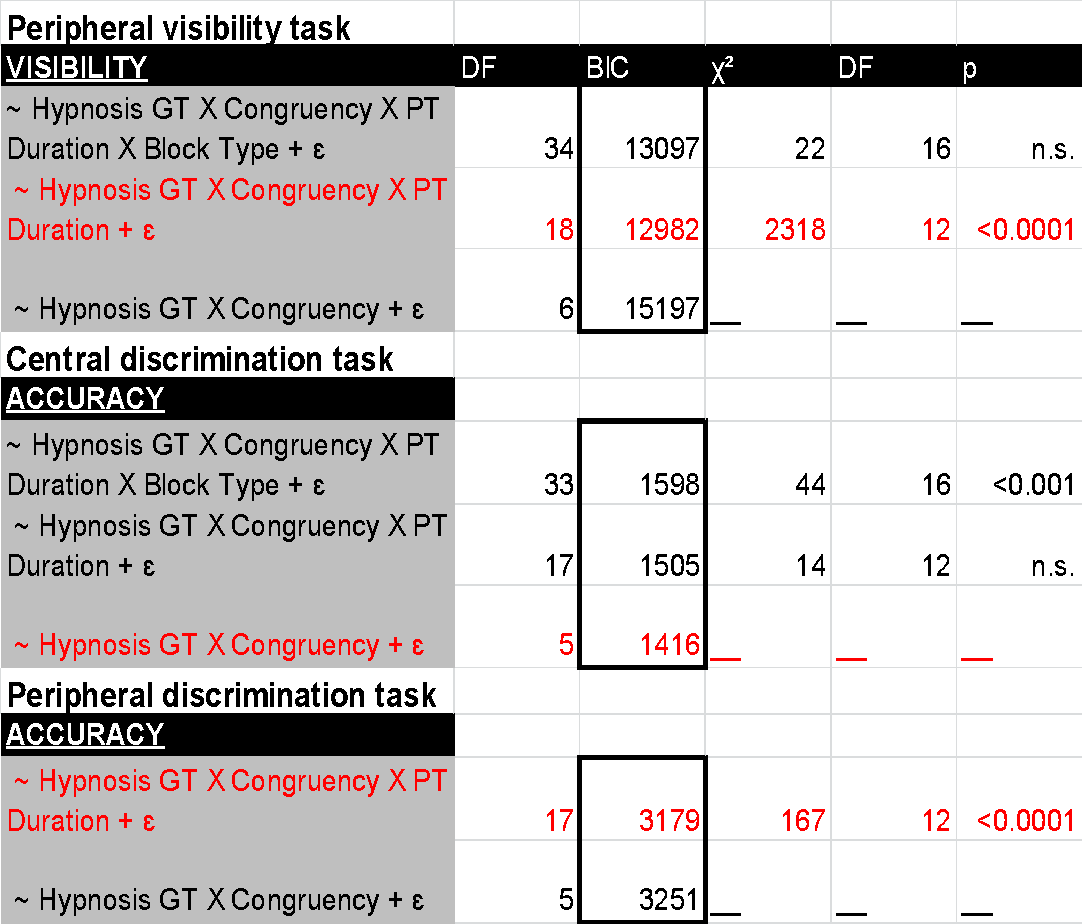
**

**Table ST 1: Full list of tested models.** Selection between different models was performed through likelihood ratio tests and by computing for each model an approximation of its Bayesian Information Criterion (BIC). We selected the models with the lowest BIC, indicated in red. For each analysis, the last line of the table host the simplest model, which was taken as reference.

*Labels for regressors: Hypnosis GT: Hypnotizability; PT duration: Peripheral Target Duration; n.s.: non-significant.*

**Figure B: Induction High vs. No Induction high contrasts for the OP block (discrimination of peripheral targets)**

Unfortunately, we were unable to use the same model structure as we had used for the first part of section 2.4.5 (High vs Low contrast) because of inescapable convergence issues. This happened because results in the whole section 2.4.5 stemmed from just one experimental block (Block OP), unlike results from other sections, which came from collapsing the trials of two blocks. This was not an issue for High vs. Low contrasts because we compensated for the lack of trials with the amount of participants (section 2.4.5, first part). Sadly, we could not do the same for Induction vs No Induction contrasts due to No Induction group n=7, a number not high enough to fit a model with Peripheral Target Duration as a fixed effect given the latter variable’s range (ass accuracy extended from 0 to 1, and PTD from 17 to 84). Now, 1) in order to privilege reproducibility, and 2) not to introduce further levels of complexity to the statistical analyses as requested in the main body of the paper, we have decided to leave this issue for the SM and “force” the fit the model by changing the Peripheral Target Duration predictor for a different factor altogether: Liminality (levels Subliminal, Supraliminal).

Our analyses showed that the accuracy differences between Induced and Non Induced Highs rose with stimulus energy (No Induction > Induction, interaction Peripheral Target Duration x Induction χ²=7.5, DF=1, p< 0.001). This is both coherent with the sense of the hypnotic suggestion and with the idea that poorer subjective view afforded participants a poorer objective performance. Of course, we are well aware the results from this fit cannot be directly compared to those of the High vs Low contrast of Study 1.


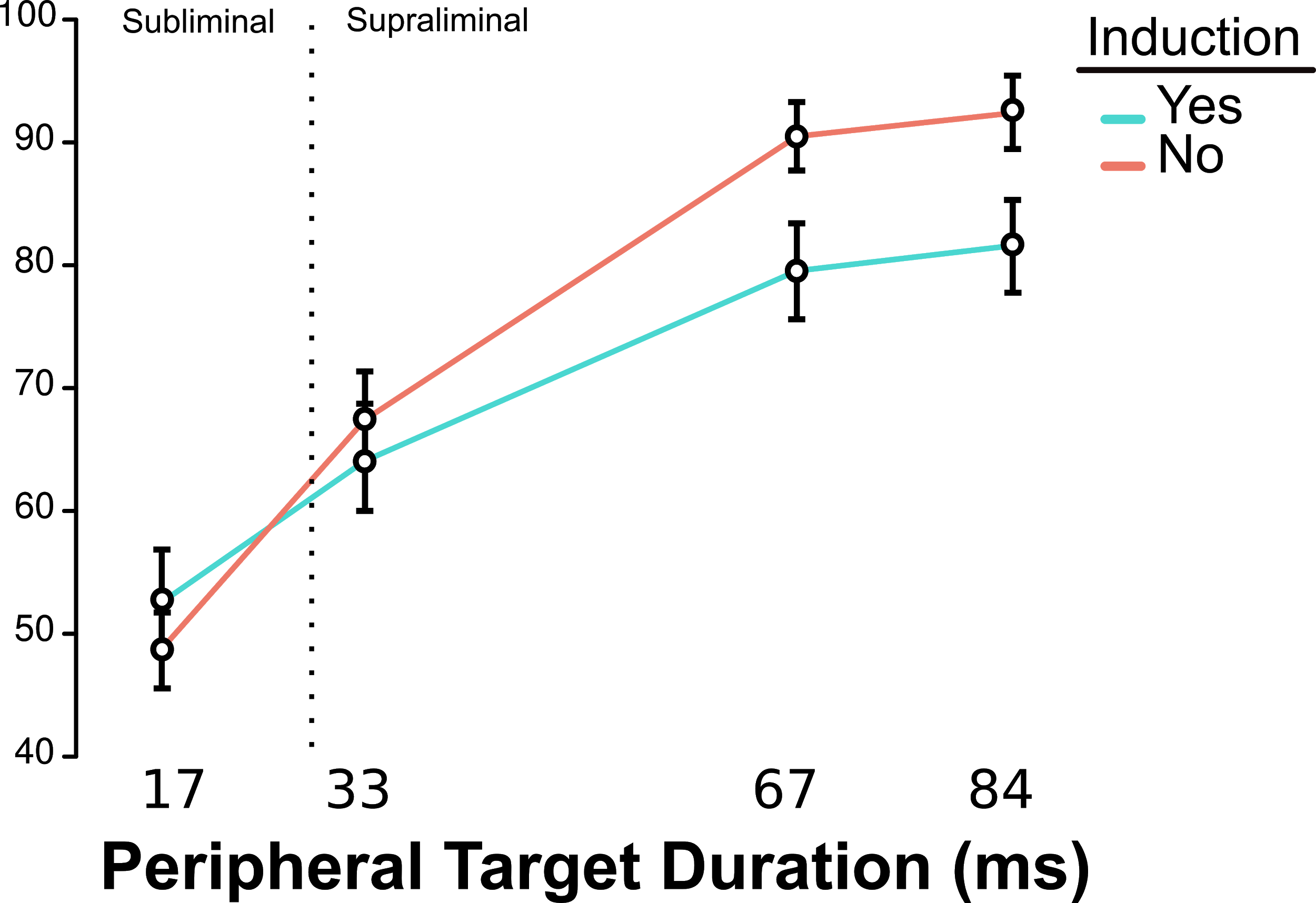


**Figure C: Interaction between Liminality and Hypnotizability for hypnotized participants (discrimination of peripheral targets)**


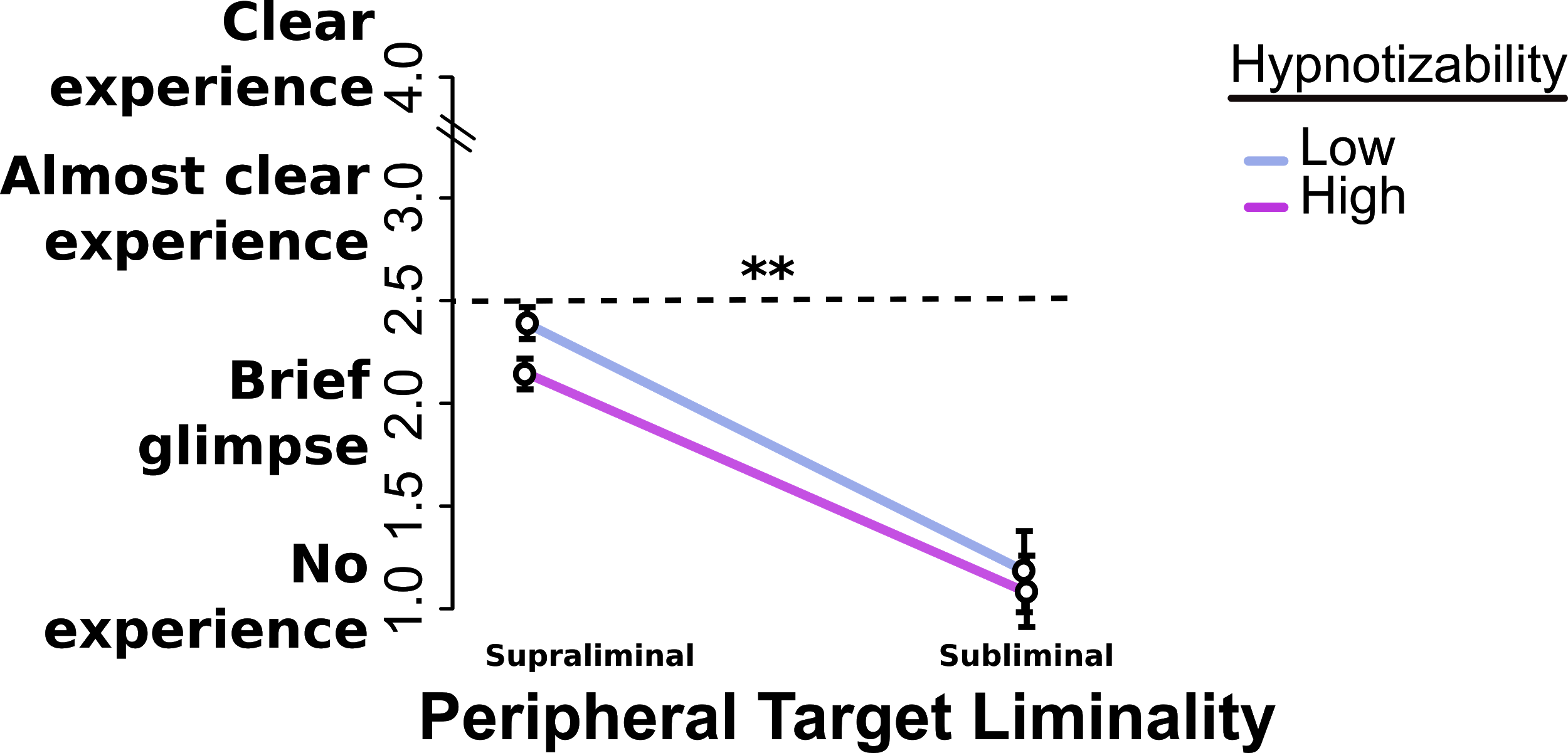


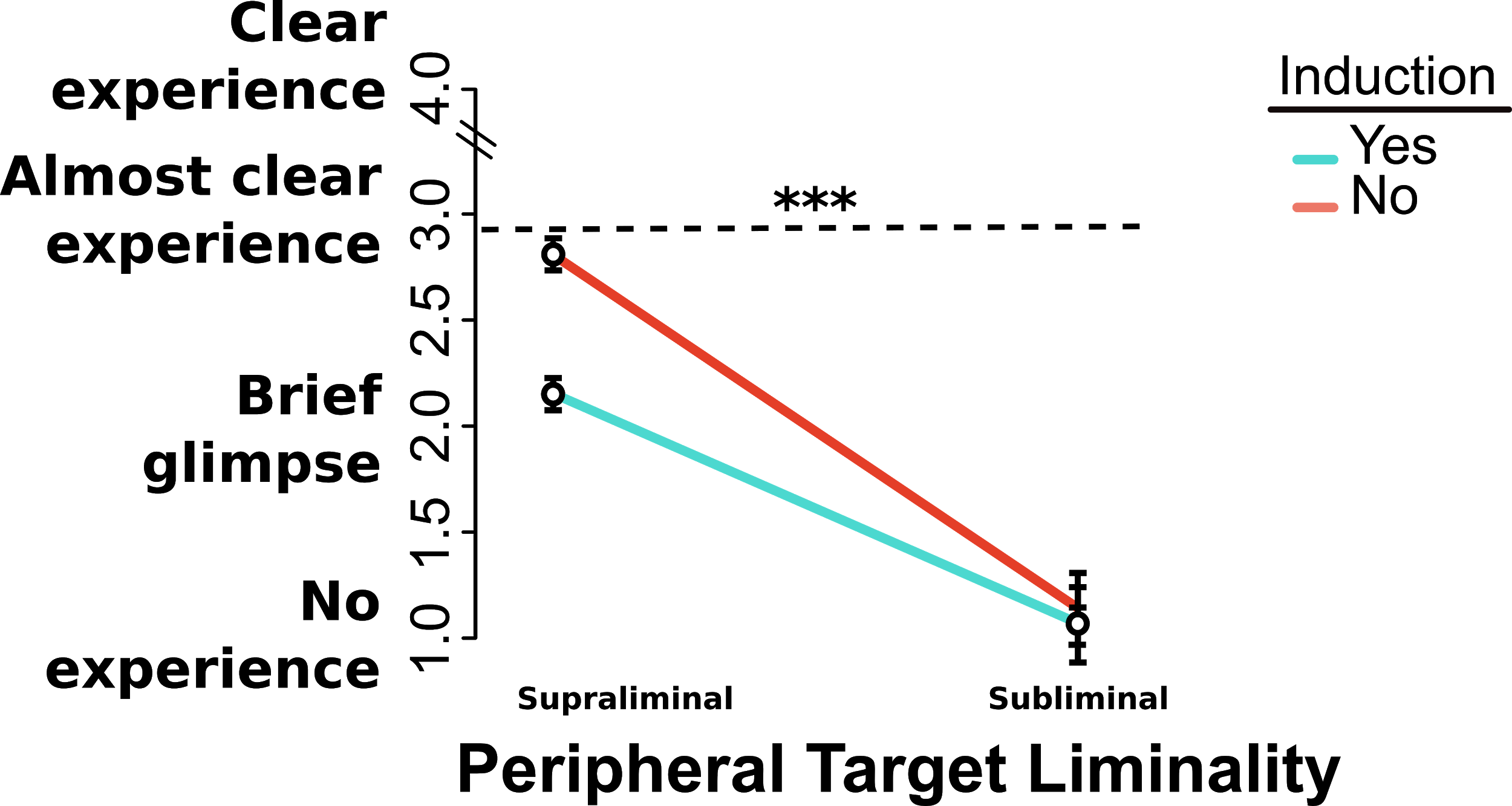


On these figures we divided our peripheral stimuli into two categories, “Subliminal” and “Supraliminal”, and found a Hypnotizability X Liminality interaction in the same direction as our reported findings for Study 1 (p < 0.01), and a Induction X Liminality interaction in the same direction for Study 2 (p < 0.0001).

**2.9.2 Annex B: Hypnotic Induction and Suggestion (Hypnotic “Balint Syndrome” inattention suggestion, based on the Harvard induction)**

**Participant handling**

Participants were greeted by the first author, who was also responsible for the hypnosis procedure. The first author remained uninformed of the hypnotic susceptibility of the participants until after the test, when the moment came for a post-test interview. Responses to participants’ questions and the interactions with them were scripted as much as possible, and discussions about the participants’ experience at the HGSHS:A screening avoided until after the test. Before starting the procedure, participants were informed again that their response, if any, to the procedure had no impact on their monetary compensation. It was also clearly stated to participants from testing session 1 that the experimenters did not expect them to express any particular degree of susceptibility to hypnotic suggestion, but that faking a response to the hypnotic procedure would be “pretty evident to any trained hypnotizer” and, while it would not impact their payment, it would lead to the experimenters having to discard the collected data and that “that would be a shame”. After signing the written consent, participants proceeded to start with the training phase, after which they received the hypnotic induction.

**Induction**

**Stage 1**

*(Merci d’être venu, et merci de travailler avec nous.) Vous avez jusqu’à présent bien travaillé durant la première partie de cet expérience, et maintenant il est temps de passer à la partie suivante. Oui, comme vous pouvez probablement l’imaginer maintenant, nous allons commencer avec l’induction hypnotique. C’est une procédure que vous connaissez bien maintenant. Vous connaissez l’effet que ça porte sur votre esprit et sur votre corps, car pendant le test de l’échelle dont vous avez déjà participé vous avez bien ressenti la trance hypnotique. Tout ce qu’on va faire maintenant c’est simplement de rentrer dans cet état que vous connaissez déjà, qui vous est familier. Nous allons à nouveau le revivre avec toutes les sensations plaisantes qui en font partie. C’est bien pour vous maintenant de commencer à vous souvenir de cette occasion durant laquelle vous avez été hypnotisé chez nous, alors que vous vous préparez pour cette expérience. Très bien. Permettez-vous de commencer à tout revivre maintenant alors que nous nous préparons à commencer. Excellent.*

**[**(Thank you for coming, and for working with us.) You have up until now worked very well during the first part of this experiment, and now it’s time to move on to the next stage. Yes, as you may probably imagine, we shall now start a hypnotic induction. This is a procedure that you now know well. You know the effect it has on your mind and body, since during the screening test you have already participated of you already felt quite well the hypnotic process. All we will be doing now is simply going back into this state you already know, that you are familiar with. We will re-live it, together with all the fine sensations that come with it. It’s ok for you now to start remembering that time in which you were hypnotized with us, while you’re getting ready for this experience. That’s great. Allow yourself to start re-living the whole thing while we get ready to start. Excellent.**]**

*Je veux que vous vous adossiez confortablement et que vous reposiez vos mains sur vos genoux. C’est ça. Reposez vos mains sur vos genoux. S’il vous plait, regardez maintenant vos mains et choisissez un point sur l’un d’entre elles. Le point que vous choisissez n’est pas important, choisissez tout simplement un point sur lequel vous vous concentrer. S’il vous plait, regardez directement ce point pendant que vous suivez ma voix, et concentrez-vous sur les instructions que je suis sur le point de vous donner. Elles vont vous aider à vous relaxer et à progressivement atteindre un état d’hypnose. Peu importe le point que vous choisissez, choisissez simplement un point sur lequel se concentrer. Je vais faire référence à ce point que vous avez choisi comme «cible ». C’est parfait . . . mains relaxées . . . regardez directement la cible. Je suis sur le point de vous donner quelques instructions qui vous aideront à vous relaxer et à rentrer graduellement dans un état d’hypnose. Simplement relaxez-vous et mettez-vous à l’aise. Je veux que vous regardiez constamment la cible et pendant que vous gardez vos yeux sur elle, écoutez ce que je dis. Votre capacité à être hypnotisé dépend en partie de votre volonté à coopérer, et en partie de votre capacité à vous concentrer sur la cible et sur mes mots. Vous avez déjà démontré que vous étiez coopératif en venant ici aujourd’hui, et avec votre coopération je peux vous aider à être hypnotisé. Vous ne pouvez être hypnotisé que si vous le voulez, je suppose que vous le voulez et que vous faites au mieux pour coopérer en vous concentrant sur la cible et en écoutant mes mots, en laissant arriver les sensations nouvelles qui vont se produire. Simplement laissez-le arriver. Et si vous faites très attention à ce que je vous dis, et que vous pensez à ce que je vous demande de penser, vous allez facilement ressentir ce que c’est qu’être hypnotisé. Comme vous le savez maintenant, l’hypnose est un phénomène naturel. Il est probable que vous allez commencer à vous souvenir de la ou les expériences hypnotiques précédentes… la manière dont vous les avez ressentie, la manière dont vous les ressentez… Ça c’est bien, vous pouvez vous permettre de tout ressentir à nouveau. Revivez-tout cela en suivant mes instructions. Relaxez-vous, tout simplement. Soyez confortable. Gardez vos yeux sur la cible. Regardez-la aussi constamment que vous le pouvez. Si vos yeux s’en écartent, ce n’est pas important . . . faites juste revenir vos yeux sur la cible.* ***En fait, vous allez découvrir que vous devenez de plus en plus capable de fixer vos yeux sur la cible au point qu’ils deviennent complètement immobiles, que vous regardez la cible et que la cible, et rien d’autre. Très très bien.***  *Après un moment, vous allez peut être trouver que la cible devient floue, ou peut-être qu’elle bouge, ou encore qu’elle change de couleur. Ce n’est pas important.* ***Ça n’a pas d’effet sur votre capacité extraordinaire de fixer votre attention et votre regarde sur la cible, au point que tout ce qui se trouve à côté de la cible deviens peut être flou ou même invisible. Ceci est tout à fait normal, et même utile car ce qui se passe autour de la cible n’a aucun intérêt pour vous.*** *Si vous somnolez un petit peu, ça aussi c’est normal. Peu importe ce qui se passe, laissez-le se passer, et continuez à fixer la cible pendant un moment. Il viendra un moment, ou vos yeux seront tellement fatigués, ils seront tellement lourds, que vous ne serez plus capable de continuer à les garder ouverts, et ils se fermeront, peut-être de manière involontaire. Quand cela arrivera, laissez-le se produire. Très très bien.*

**[**I would like you to lie comfortably on your chair and to rest your hands on your lap. That’s it. Lay your hands on your lap. Please, look down to your hands now and pick a spot upon which to concentrate. Whatever spot you may choose is not important, simply choose a spot on either one of your hands and concentrate on it. Please, look straight at the spot while you follow my voice, and concentrate on the instructions that I’m about to give you. They will help you to relax and to progressively achieve a state of hypnosis. It doesn’t matter which spot you choose, just choose a spot to concentrate on. I shall now address that spot you have selected as “the target”. That’s perfect… Hands relaxed… look directly at the target. I’m about to give you some instructions that will help you relax and gradually achieve a state of hypnosis. Simply relax and get comfortable. I would like you to keep looking at the target, and while you keep your eyes on it, listen to what I have to say. Your ability to be hypnotized depends partially of your will to cooperate, and partly of your ability to concentrate on the target and on my words. You have already shown that you were willing to cooperate by coming here today, and with your cooperation I can help you become hypnotized. You cannot be hypnotized unless you want it, and I suppose that you want it and that you are doing your best to collaborate with us and to concentrate on the target and on my words, to welcome all of the new sensations that may soon take place. Just let them take place. And if you really pay attention to what I am saying, and you think about what I’m asking you to think about, you will easily feel what being hypnotized is like. As you now know, hypnosis is a natural phenomenon. It is likely that you may start remembering any or all of your previous hypnotic experiences… the way they felt, what it feels like right now… That’s ok, you can allow yourself to feel all this all over again. You can re-live the experience while you listen to my instructions. Relax completely. Be comfortable. Keep your eyes on the target. Look at it as constantly as you can. If your eyes stray away from it, it’s ok… just bring them back on the target. **You will actually start to notice that you become more and more capable of fixating your eyes on the target, to the point of rendering them completely still, of looking at the target and nothing but the target. Very good!** After a little while, you may find that the target becomes blurry, or that it moves, even… or that it changes color. This is ok. **It has no effect on your extraordinary capacity to fixate your attention and your gaze upon the target, to the point that anything around and outside of the target becomes maybe blurry or totally invisible. This is perfectly normal, and even useful to you, as nothing outside the target is of any interest to you at the moment.** If you feel slightly tired, this is normal too. Whatever may happen, just let it happen, and keep looking at the target. The moment will come in which your eyes will be so tired, so heavy, that you may become unable to keep them open, and they will close, maybe by themselves. When this happens, and it will, just let it happen. Very good, that’s perfect.**]**

*Vous allez découvrir que vous pouvez porter votre attention sur votre corps en lui permettent de trouver le confort tout en se relaxant. Maintenant, relaxez tous les muscles de votre corps. Relaxez les muscles de vos jambes. . . Relaxez les muscles de vos pieds. . . Relaxez les muscles de vos bras. . . Relaxez les muscles de vos mains . . . de vos doigts . . . relaxez les muscles de votre cou, de votre poitrine . . . relaxez tous les muscles de votre corps. Relaxez-vous de plus en plus, de plus en plus. Relaxez-vous complétement. Relaxez-vous complétement. Relaxez-vous complétement. Très très bien.*

*Votre vision devient floue, vos paupières clignent et vos yeux sont peut-être déjà fermés. Très très bien. Il est très agréable de fermer vos yeux, de vous relaxer complétement, et d’écouter, ma voix qui vous parle. Vos yeux sont fermés maintenant, et s’ils ne le sont pas, ils devraient bientôt se fermer d’eux-mêmes. Mais il n’est pas nécessaire de continuer à les forcer. Même si vos yeux ne se sont pas fermés complétement pour l’instant, vous vous êtes bien concentré sur la cible, et vous êtes confortable.*

**[**You will realize that you can direct your attention towards your own body, allowing it to find a deeper comfort as it relaxes. Now, relax all the muscles in your body. Relax the muscles of your legs… Relax the muscles of your feet… Relax the muscles of your arms… Relax the muscles of your hands… of your fingers… relax the muscles of your neck, of your chest… relax every muscle of your body. Relax more and more, more and more. Relax completely. Relax completely. Relax completely. Very good.

Your vision becomes blurry, your eyes blink and are maybe already closed. Very good. It is quite pleasant to close your eyes, to relax completely, and to listen to my voice talking to you. Your eyes are now closed, and if they aren’t, they should close by themselves soon enough. But it’s not necessary to continue to force them. Even if your eyes aren’t completely closed right now, you are very well concentrated on the target, and you are comfortable.**]**

**Stage 2**

*Vous êtes maintenant confortablement relaxé, et vous allez vous relaxer encore plus. Et encore plus. Vos yeux sont maintenant fermés. Vous allez garder vos yeux fermés, jusqu’à ce que je vous dise de vous éveiller. . . . Vous vous sentez confortable. Continuez simplement à écouter ma voix. Faites très attention à elle. Gardez vos pensées sur ce que je dis, écoutez simplement. Vous allez devenir beaucoup plus confortable. Bientôt vous serez toujours confortable, mais vous continuerez à m’écouter. Vous ne vous éveillerez pas avant que je vous le dise. Je vais maintenant commencer à compter. À chaque chiffre, vous allez évoluer vers ce confort profond. Un état dans lequel vous serez capable de faire tout sorte de choses que je vous demanderez de faire. Un—vous allez vers cette état profonde. . . deux— de plus en plus profonde et confortable. . . trois—quatre—de plus en plus, de plus en plus confortable. . . cinq—six—sept--vous glissez agréablement dans cette état… Ne faites attention qu’à ma voix, et portez votre attention sur les éléments auxquelles je vous demanderai de faire attention. Je voudrais que vous continuez à faire attention à ma voix et aux choses que je vous dis. . . huit—neuf—dix—onze—douze—De plus en plus d’attention, toujours profondément confortable—treize—quatorze—quinze—même si vous êtes profondément confortable vous pouvez clairement m’entendre. Vous allez toujours m’entendre, peu importe à quel niveau de profondeur vous sentez que vous êtes arrivé . . . seize—dix-sept—dix-huit—profondément, agréablement confortable, tonique mais confortable. Vous allez ressentir beaucoup des sensations que je vais vous demander de ressentir. . . Dix-neuf, Vingt. Profondément confortable! Vous allez ressentir ce que je vais maintenant décrire.*

**[**You are now comfortably relaxed, and you will relax even more. And even more. Your eyes are now closed. You shall keep them that way, up until I tell you come out of it… You are feeling comfortable. Just continue listening to my voice. Pay very good attention to it. Keep whatever you may think about what I’m saying to yourself, and just listen. You will get even more comfortable. Soon you’ll be even more comfortable but yet you shall continue to listen to me. You will not come out of it before I tell you so. I will now start counting. At each number, you will continue to evolve towards this profound comfort. A state in which you will be fully capable of doing all sort of things I shall ask you to do. One- you move forward towards this profound state… two- more and more comfortable… three- four- more and more, more and more comfortable… five- six- seven- you glide gently into this state… Pay attention to my voice and nothing else, and to the elements I ask you to pay attention to. I would like you to continue to pay attention to my voice and to the things I say… eight- nine- ten- eleven- twelve, More and more attention, always deeply comfortable… thirteen- fourteen- fifteen- even if you’re deeply comfortable you can listen to me clearly. You will continue to listen to me, regardless of the level of depth you feel you have achieved… sixteen- seventeen- eighteen- deeply, nicely comfortable, aware but comfortable. You will feel plenty of sensations that I will ask you to feel… nineteen- twenty. Deeply comfortable! You will now feel what I’m about to describe.**]**

Poshypnotic suggestion and de-induction:

**Stage 1**

*Comme vous le savez très bien, vous êtes ici aujourd’hui pour participer à une expérience. Suivre les instructions en fait va vous aider à rester dans l’état de transe. Vous devez savoir qu’un individu peut parfaitement ouvrir ses yeux et s’engager dans toutes sortes de tâches, en continuant toujours à vivre profondément les effets de sa trance hypnotique. En fait, en suivant les instructions, vous trouverez, que vous le réalisiez ou non, que l’effet des suggestions donnés pendant l’hypnose devient encore plus consolidé. Plus vous travaillez, plus vous suivez mes instructions, plus vous appréciez l’expérience plaisante de la transe. Et ça c’est très bien.*

*Vous avez devant vous, comme vous le savez déjà, un clavier et un écran. Sur l’écran vous allez vérifier que pendant la tâche que nous allons vous demander de faire bientôt il y a des images, des lettres et chiffres, qui s’affichent dans l’écran. Aussi, notamment, comme je vous ai montré déjà, une ellipse qui s’affiche au centre même de l’écran. Quelque chose de très intéressant va se passer avec cette ellipse.*

***Au moment de commencer la tâche, vous allez suivre mes instructions et fixer votre attention et votre regarde sur l’ellipse. Et vous allez le faire tellement, que vos yeux vont rester immobiles sur cette ellipse. Vos yeux vont rester immobiles sur cette ellipse et votre attention va se concentrer exclusivement sur cette ellipse et son contenu. Au point qu’il n’y a rien autour de cette ellipse, tout autour de l’ellipse est invisible : il n’y a rien autour de l’ellipse, et même si quelqu’un voudrait afficher quelque chose sur l’écran autour de l’ellipse vous allez l’ignorer au point de ne pas le voir. Oui. Pendant que vous regardez l’écran vous allez voir exclusivement l’ellipse et son contenu. Et ça serait très bien pour vous. Sans doute vous allez percevoir l’écran différemment de comment vous le percevez d’habitude, et ça c’est très bien. Que vous vous souvenez des instructions que je viens de vous donner ou vous les oubliez, ou que vous les ressentez de manière consciente… peu importe, car l’effet va se passer quand même grâce à votre travail active et inconsciente. Plus vous regardez l’écran, plus cet effet devient fort et consolidé. Plus votre attention et vos préférences se concentrent exclusivement sur l’ellipse du centre et son contenu.***

**[** As you know all too well, you are here today to participate in an experiment. Following the instructions will actually help you stay in a trance state. You must know that a person can perfectly open her eyes and engage on all sorts of tasks, while still experiencing the profound effects of her hypnotic trance. Actually, following the task instructions, whether you realize it or not, will consolidate the effects of any suggestion given to you. The more you work, the more you follow my instructions, the more you experience the pleasant experience that is the trance. And that’s great.

You have now in front of you, as you already know, a keyboard and a screen. On the screen you will see that during the task that we will ask you to complete some images will show up, letters and numbers… they will all appear on the screen. Also, as I have already shown you, an ellipse lays in the very center of the screen. Something very interesting is about to happen with this ellipse.

**As you start the task, you will follow my instructions and fixate your attention and your gaze on the ellipse. And you’ll do this so much so, that your eyes will stand motionless on the center of this ellipse. They will stay motionless on this ellipse, and your attention will concentrate exclusively on the ellipse and its contents. To the extent that there’s nothing outside and around this ellipse, everything outside of it is invisible: there’s nothing around the ellipse, and even if anyone tries to display something outside of the ellipse you will ignore it to the point of not seeing it. Yes. While you look at the screen you will focus exclusively on the ellipse and its contents. And that will be just perfect for all of us. Surely you will perceive the screen differently from how you normally perceive it, and that’s also very good. Whether you’ll remember these instructions I have just mentioned or you’ll just forget them, or that you’ll keep them in your mind unconsciously… it’s of no consequence, since the effect will be there anyhow due to your active and unconscious work. The more you look at the screen, the more this effect becomes strong and consolidated. The more your attention and your preferences concentrate exclusively on the central ellipse and its contents.]**

**Stage 2**

*Demeurez profondément détendu et faites bien attention à ce que je vais vous dire maintenant. Dans un instant, je vais commencer à compter à rebours de 20 jusqu’à 1. Vous allez graduellement vous éveiller, mais pendant la plupart du temps où je vais compter, vous allez encore demeurer dans l’état où vous êtes maintenant. Au moment où je dirai cinq, vous allez ouvrir vos yeux, mais vous ne serez pas complètement éveillé.* ***DÈS QUE VOUS VOUS RETROUVEREZ DEVANT L'ÉCRAN ça va se faire de soi-même : il n’y a rien autour de cette ellipse, tout autour de l’ellipse est « très invisible ».*** *Lorsque je dirai un, vous allez être complètement vigilant, dans votre état normal d’éveil, et* ***DÈS QUE VOUS VOUS RETROUVEREZ DEVANT L’ECRAN, ça va s’installer : il n’y a rien autour de cette ellipse, tout autour de l’ellipse est « très invisible ».***

**[**Remain deeply relaxed and pay attention to what I’m about to tell you now. In a moment, I’ll start counting backwards from 20 to 1. You will gradually come out of it, but for the most of this countdown, you will still remain in the state you are now. When I say five, you will open your eyes, but you will not be fully out of it. **AS SOON AS YOU FIND YOURSELF IN FRONT OF THE SCREEN, it will happen on its own: there’s nothing around the ellipse, everything around the ellipse is “very much invisible”.** As I say one, you will be completely vigilant, out of it, in your normal wake state, and **AS SOON AS YOU FIND YOURSELF IN FRONT OF THE SCREEN, it will happen on its own: there’s nothing around the ellipse, everything around the ellipse is “very much invisible”.]**

**Stage 3**

*Je vais maintenant commencer à compter à rebours en partant de vingt, et à cinq, pas avant pas après, vous allez ouvrir vos yeux, mais vous ne serez pas complètement éveillé avant que je dise un, vers* ***le vide… à l’extérieur de l’ellipse…*** *. Je vais maintenant commencer à compter à rebours en partant de vingt, et à cinq, pas avant, vous allez ouvrir vos yeux, mais vous ne serez pas complètement éveillé avant que je dise un. À un, vous serez éveillé... Prêt, maintenant (lentement et après plus rapidement vers la fin) : 20-19-18-17-16-15-14-13-12-11-10-la moitié-9-8-7-6-5-4-3-2-1. Éveillez-vous ! Complètement éveillé ! Confortable et tonique. Très très bien.*

**[**I will now start to count backwards starting from twenty, and on five, not before, not afterwards, you will open your eyes, but you will not be completely out of it until I say one, towards the void outside of the ellipse… I will now start counting starting from 20, and on five, you will open your eyes, but you will not be completely out of it before I say one. On one you will be fully out of it…. Ready, go (slowly and progressively faster towards the end) 20-19-18-17-16-15-14-13-12-11-10-la moitié-9-8-7-6-5-4-3-2-1. Fully out of it! In your normal wake state! Comfortable and performant. Very very good.**]**

**Suggestion (without induction)**

**Stage 1**

*Vous avez devant vous, comme vous le savez déjà, un clavier et un écran. Sur l’écran vous allez vérifier que pendant la tâche que nous allons vous demander de faire bientôt il y a des images, des lettres et chiffres, qui s’affichent dans l’écran. Aussi, notamment, comme je vous ai montré déjà, une ellipse qui s’affiche au centre même de l’écran. Nous allons vous suggérer quelque chose de particulier par rapport a cette ellipse.*

***Au moment de commencer la tâche, je voudrais bien que vous fixez votre attention et votre regarde sur l’ellipse. Et si possible je voudrais que vous puissiez le faire au point que vos yeux restent immobiles sur cette ellipse. Vos yeux vont rester immobiles sur cette ellipse et votre attention va se concentrer exclusivement sur cette ellipse et son contenu. Au point que vous pourriez avoir l’impresion de qu’il n’y a rien autour de cette ellipse, et de que tout autour de l’ellipse est invisible : il n’y a rien autour de l’ellipse, et même si quelqu’un voudrait afficher quelque chose sur l’écran autour de l’ellipse vous allez l’ignorer au point de ne pas le voir. Oui. Pendant que vous regardez l’écran vous allez voir exclusivement l’ellipse et son contenu. Et ça serait très bien pour vous. Sans doute vous allez percevoir l’écran différemment de comment vous le percevez d’habitude, mais ça serait très bien. Ce n’est pas necessaire de beaucoup réfléchir aux instructions que je viens de vous donner ou les ressentir constamment de manière consciente… Naturellement vous trouverez que regarder l’écran va bien vous aider pour que votre attention soit fortement située sur l’ellipse. Plus votre attention et vos préférences se concentrent exclusivement sur l’ellipse du centre et son contenu.***

**[** You have now in front of you, as you already know, a keyboard and a screen. On the screen you will see that during the task that we will ask you to complete some images will show up, letters and numbers… they will all appear on the screen. Also, as I have already shown you, an ellipse lays in the very center of the screen. I am about to suggest that you adopt a particular stance regarding this ellipse, one that you may find intriguing.

**As you start the task, I would like you to follow my instructions and fixate your attention and your gaze on the ellipse. I’d like you to do this so much so, that your eyes may feel motionlessly posed on the center of this ellipse. Let them stay motionless on this ellipse, and your attention will concentrate exclusively on the ellipse and its contents. To the extent that at times it may seem like there’s nothing outside and around this ellipse, as if everything outside of it were invisible: there’s nothing around the ellipse, and even if anyone tries to display something outside of the ellipse you might find yourself ignoring it to the point of not seeing it. Yes. While you look at the screen you will focus exclusively on the ellipse and its contents. And that will be ok. Surely you will perceive the screen differently from how you normally perceive it, and that’s also very good. You don’t need to keep consciously thinking about these instructions I have just mentioned. You may find that the more you look at the screen, the easier it gets for this effect to become stronger and consolidated. And your attention and your preferences may concentrate exclusively on the central ellipse and its contents.]**
